# Association of Early and Late Postnatal Serum Lactate/Albumin Ratio and Retinopathy of Prematurity in Preterm Infants: A 2 Time Point Comparative Study

**DOI:** 10.5152/eurasianjmed.2026.261457

**Published:** 2026-08-20

**Authors:** Büşra Çalışkan, Dilara Korkut, Mustafa Kara, Kadir Şerafettin Tekgündüz

**Affiliations:** 1 Department of Ophthalmology, Atatürk University Faculty of Medicine, Erzurum, Türkiye; 2 Department of Neonatology, Atatürk University Faculty of Medicine,Erzurum, Türkiye

**Keywords:** Albumin, biomarker, lactate, retinopathy of prematurity, the lactate-albumin ratio

## Abstract

**Background:**

One easily accessible compound index that shows the metabolic and inflammatory status of critically ill patients is the lactate-albumin ratio (LAR). This study set out to evaluate the connection between LAR and retinopathy of prematurity (ROP) in premature infants.

**Methods:**

The investigation was conducted retrospectively on 150 premature infants born before 34 weeks who underwent screening for ROP in the neonatal intensive care unit. Demographic and clinical data, as well as ROP examination findings, were recorded for all participants. For the first 24 h following delivery (early phase) and at 34-36 weeks’ postmenstrual age (late period), serum lactate and albumin values were measured separately. For each of these measurements, the lactate/albumin ratio was calculated. Mann– Whitney *U-*test, logistic regression, correlation analyses, together with receiver operating characteristic (ROC) analysis were performed.

**Results:**

: In infants who developed ROP, both the LAR calculated in the early phase (1.16 ± 0.56, *P* = .03) and the LAR calculated in the late period (0.53 ± 0.19, *P* = .02) were found to be significantly higher than those without ROP. In post hoc analyses based on disease severity, LAR values were significantly higher in the treatment-requiring ROP group (*P* = .02). In logistic regression, late-period LAR showed a borderline but statistically non-significant association with ROP development (OR = 11.05; 95% CI = 0.97-125.9; *P* = .053).

**Conclusion:**

Lactate-albumin ratio values were associated with ROP development and disease severity in premature infants. However, LAR was not demonstrated to be an independent predictor of ROP in the present study. As a composite index reflecting nutrition, inflammation, tissue oxygenation, and metabolic stress, LAR may provide complementary information regarding systemic vulnerability in premature infants rather than serving as a standalone predictive or screening biomarker. Further prospective and multicenter studies are needed to clarify its potential role in ROP risk assessment.

**Main Points**

- Compared to premature infants who did not develop retinopathy of prematurity (ROP), those who did had a significantly higher lactate/albumin ratio (LAR).
- Both early-postnatal and late-postnatal LAR values were associated with ROP, suggesting that LAR may reflect ongoing metabolic and inflammatory stress related to disease development.
- Late-period LAR values were significantly higher in infants requiring treatment, indicating a possible relationship between LAR and disease severity.
- Although LAR showed limited discriminatory performance in ROC analysis, it may provide complementary information regarding systemic vulnerability in preterm infants.
- Lactate-albumin ratio should be interpreted as an accessible complementary marker of systemic vulnerability rather than as a standalone predictor of ROP.

## Introduction

Despite improvements in neonatal care, retinopathy of prematurity (ROP), a proliferative condition of the microvasculature of the retina, remains one of the leading causes of pediatric vision loss on a worldwide scale.¹ Disturbances in oxygen homeostasis during the perinatal period may contribute to hypoxic–ischemic stress, leading to the release of pathological angiogenic factors and abnormal retinal vascularization.^1^ According to epidemiological research, premature infants with reduced gestational age and birth weight have a higher prevalence and severity of ROP.^2^

Comprehensive ROP screening is associated with quality neonatal care. However, a balance is needed between correctly identifying the high-risk group and reducing the workload associated with unnecessary examinations of premature infants.^3^

Lactate functions as an indicator of tissue hypoxia, signifying abnormal perfusion. It is a proangiogenic molecule produced as the end product of glucose glycolytic metabolism. Lactate causes the production of vascular endothelial growth factor (VEGF) by facilitating the combination of hypoxia-inducible factor-1α (HIF-1α) with HIF-1β.^4^ It is used as a guide for the prognosis of various diseases in adults and children.^5^^,^^6^

Serum albumin functions as a nutritional indicator and a negative acute-phase reactant in inflammatory states. Reduced albumin concentrations have been related to higher mortality among premature infants.^7^

Recently, the lactate-albumin ratio (LAR) has been regarded as a prognostic indicator that integrates metabolic and inflammatory changes and shows a close relationship with the severity of critical illness.^8^ Wang and colleagues initially described LAR to be a possible marker of mortality and organ failure. Elevated LAR has been linked to increased multiple organ dysfunction syndrome (MODS) and mortality in individuals with sepsis.^9^ Since then, this ratio has been used as a predictive biomarker in several critical illnesses.^10^^,^^11^ LAR is increasingly being recognized as a predictive tool for severely ill pediatric patients. High LAR levels have been shown to be related to increased rates of MODS and mortality relative to lactate and albumin assessed separately.^8^ LAR and 28-day all-cause mortality in pediatric sepsis patients have been found to be strongly correlated.^12^ This pathophysiology of tissue hypoxia and sepsis-related organ failure is significantly influenced by microcirculatory dysfunction.^13^

Oxygen imbalance and retinal hypoxia cause ROP, a 2-phase retinal vascular developmental disorder in premature infants. In the early phase, relative hyperoxia can inhibit the HIF-1α/VEGF signaling pathway, thereby halting normal vascular development; in the subsequent hypoxic phase, HIF-1α stabilization and increased VEGF levels trigger pathological neovascularization. Consequently, the HIF-1α/VEGF axis plays a fundamental role in both the vascular developmental disorder and the progressive neovascular phase of ROP.^14^

In this context, considering the central role of oxygen imbalance, retinal hypoxia, and the HIF-1α/ VEGF-mediated angiogenic response in the pathophysiology of ROP, and considering that lactate reflects hypoxic-metabolic stress whereas albumin reflects inflammation, nutritional status, and systemic disease burden, LAR may reflect the systemic stress environment associated with ROP development and progression as a composite index.

Based on this background, they aimed to examine the association of early- and late-postnatal LAR values with the development and severity of ROP in premature infants.

## Material and Methods

This retrospective study evaluated 150 premature infants who underwent ROP screening in the Neonatal Intensive Care Unit of the Faculty of Medicine at Ataturk University during February 1, 2020, and August 28, 2025. The research was performed in line with the Declaration of Helsinki and was approved by the Ataturk University Non-Interventional Clinical Research Ethics Committee (Approval date: November 28, 2025, Approval no: 821). The ethics committee decided the study does not require informed consent due to the fact that it is retrospective in nature. This work was prepared using the DeepL artificial intelligence–powered translation application solely to improve the language and edit the grammar during the preparation of the article. However, the entire content of the work was reviewed and edited by the authors after the use of artificial intelligence tools.

Every participant in the study had complete data, had undergone ROP screening, had no major malformations or chromosomal abnormalities, and had no ocular abnormalities. They were all born before 34 weeks of gestation.

A subtype of ROP known as aggressive retinopathy of prematurity (A-ROP) is distinguished by posterior localisation and prominent plus disease and differs from classic ROP in its rapidly progressing clinical features. It was excluded from the study due to the small number of patients in order to avoid unreliable statistical estimates. Clinical information about each participant’s gender, gestational age, birth weight, history of mechanical ventilation, patent ductus arteriosus (PDA), surfactant therapy, bronchopulmonary dysplasia (BPD), respiratory distress syndrome (RDS), and history of sepsis was gathered.

The International Classification of Retinopathy of Prematurity and the Early Treatment for Retinopathy of Prematurity study were used to monitor and treat preterm infants.^15^^,^^16^ Until full retinal vascularization was attained, infants who either did not develop ROP or showed spontaneous regression of ROP were monitored.

Based on the examination’s findings, the study’s participants were first split into 2 groups: those with ROP and those without ROP. Patients who developed ROP were further classified into subgroups based on disease severity: those with spontaneous regression (ROP-r) and those requiring treatment (ROP-t).

The assessment time points were set as the first 24 h after birth and 34-36 weeks’ postmenstrual age. The first 24 h were defined as the early phase, reflecting antenatal effects and early metabolic adaptation; in contrast, the 34- to 36-week postmenstrual age range was selected as the late-phase assessment point, as it coincides with the period when Phase II of ROP becomes evident, and the condition becomes clinically recognizable.

In this phase, HIF-1α/VEGF activation associated with retinal hypoxia increases, and pathological vasoproliferation may develop alongside rising retinal metabolic demands. The fact that the median age at the first diagnosis of ROP in infants in the moderate-risk group has been reported to be approximately 35.3 postmenstrual weeks further supports the clinical appropriateness of this timeframe.^17^^,^^18^

Within the initial 24-hour postnatal period (early phase; LAR1) and at 34-36 weeks’ postmenstrual age (late period; LAR2), serum lactate and albumin values were measured separately. The lactate/albumin ratio was computed for each of these measurements. LAR was computed by dividing serum albumin (g/dL) by serum lactate (mmol/L).

### Statistical Analysis

The statistical evaluation was carried out using IBM SPSS Statistics for Windows, version 20.0 (IBM Corp.; Armonk, NY, USA). All data were summarized by descriptive measures, including the mean and standard deviation. The normality of the data distribution was examined with the Shapiro–Wilk test. Variables that were not normally distributed were evaluated using the Mann–Whitney *U-*test, whereas normally distributed parameters were compared with the *t*-test. Categorical variables were assessed with chi-square tests. ROC analysis was used to evaluate the ability of LAR to discriminate ROP. ROC analysis was applied to assess the discriminatory performance of LAR for ROP. The associations between risk variables and LAR were examined using Spearman and Pearson correlation analyses. Multivariate logistic regression analysis was used to identify independent variables associated with ROP development. Statistical significance was set at *P* < .05. G*Power 3.1 was used to determine the sample size and the test power obtained for LAR means between the patient (n = 76) and control (n = 74) groups at a 2-tailed α of 0.05 level was calculated as 0.92.

## Results

The study included 150 premature infants who were admitted to the neonatal intensive care unit. Of the 150 patients, 76 were female (50.7%) and 74 were male (49.3%). ROP was present in 76 of all infants (50.7%). Spontaneous regression was observed in 44 infants with ROP (29.3%), while treatment was required in 32 (21.3%). The ROP (+) group had a mean birth weight of 976.8 ± 325 g (450-2200 g) and a mean gestational age of 27.59 ± 2.1 weeks (24-34 weeks), both of which were significantly lower than those of the control group (*P* < .001).

Albumin levels assessed in ROP (+) preterm infants during the first 24 h postnatally and at 34-36 weeks postmenstrual age (2.80 ± 0.36 g/dL, *P* < .001 and 3.04 ± 0.49 g/dL, *P* = .003, respectively) were lower than those in infants without ROP. The ROP (+) group had higher serum lactate levels during both the early and late phases, but the differences were not statistically significant.

In infants with ROP, both early-period LAR (1.16 ± 0.56, *P* = .03) and late-period LAR (0.53 ± 0.19, *P* = .02) were significantly higher than those in infants without ROP (Table 1).

Post-hoc analyses were conducted on subgroups of ROP patients classified according to disease severity. These analyses revealed significant differences in albumin levels across the groups at both time points.

Albumin levels were lower in the group that required treatment than in the groups that did not develop or regress ROP (*P* < .001). LAR values were lowest in the group without ROP development. LAR2 values were significantly higher in the severe ROP group that required treatment (*P* = .02; see Table 2).

**Figure 1:**
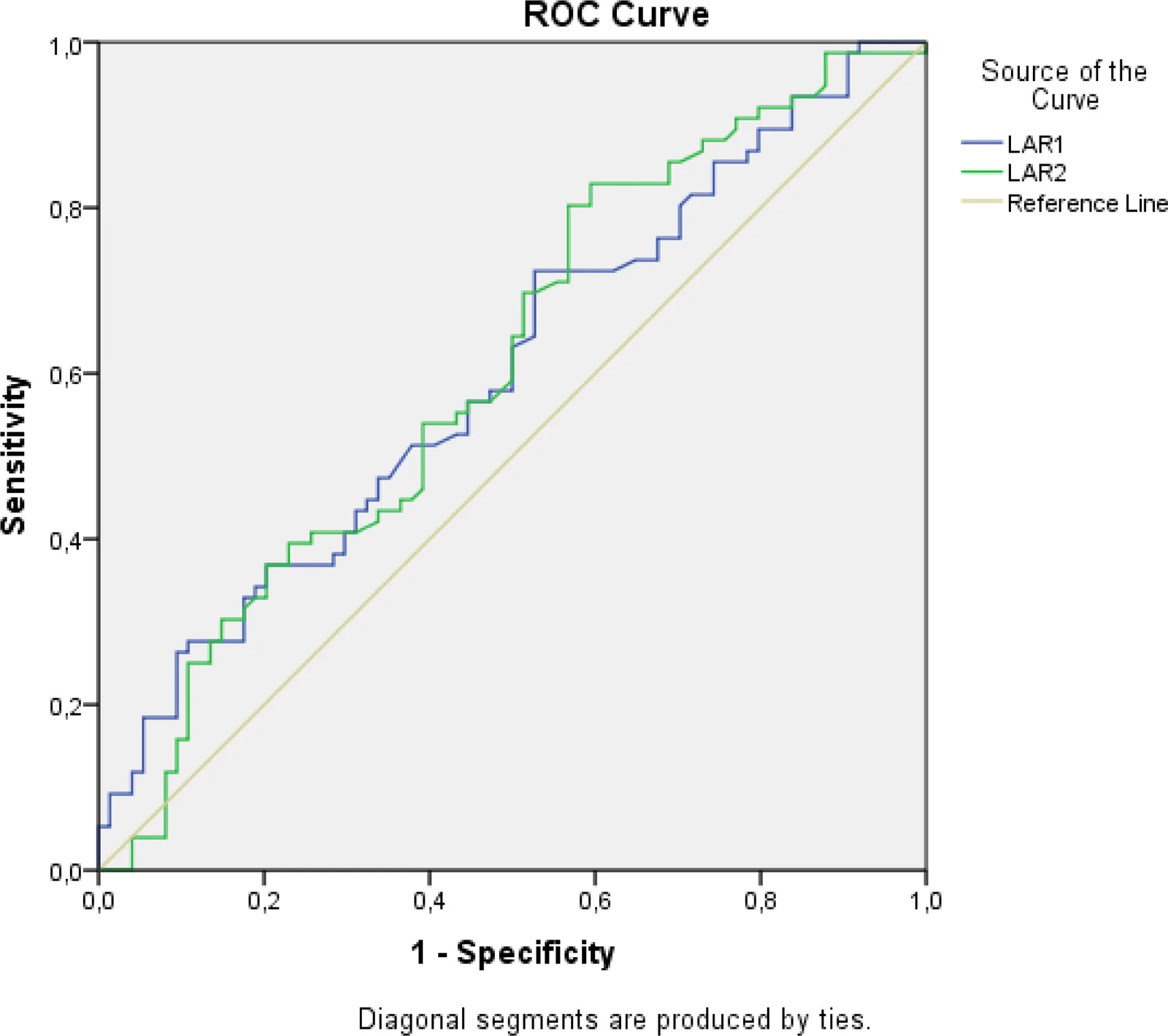
Reciever operating curve analyses of lactate-albumin ratio 1 and lactate-albumin ratio 2.

The ability of LAR to distinguish between infants with and without ROP was assessed using a receiver operating characteristic (ROC) curve analysis. LAR1’s area under the curve (AUC) was determined to be 0.601 (*P* = .033; 95% confidence interval [CI] = 0.511-0.691). The ideal cut-off point was found to be 0.81 based on the Youden index. At this cut-off point, the sensitivity was 76%, the specificity was 57%, and the Youden index was 0.33. The AUC for LAR2 was found to be 0.608 (*P* = .022; 95% CI = 0.518-0.699). The optimal cut-off value for LAR2 was found to be 0.23. At this threshold, sensitivity was 79%, specificity was 48%, and the Youden index was 0.27. Overall, these AUC values indicate limited discriminatory performance (Figure 1).

Correlation analyses revealed that LAR1 was negatively correlated with both gestational age (ρ = −.166; *P* = .04) and birth weight (ρ = −0.245; *P* = .003), and positively correlated with mechanical ventilation (ρ = 0.165; *P* = .044). Borderline negative correlations were found for LAR2 with surfactant administration (ρ = −0.157; *P* = .055), but its relationships with gestational age and birth weight were not significant.

Multivariate logistic regression analysis was conducted to identify the independent variables related to the development of ROP. The variables entered into the model were determined according to their established clinical relevance to ROP and the findings obtained from the univariate analysis. Accordingly, the model included LAR2, gestational age, birth weight, use of a mechanical ventilator, respiratory distress syndrome (RDS), patent ductus arteriosus (PDA), and surfactant therapy. Gestational age and mechanical ventilation were found to be independently associated with ROP development. LAR2 showed a borderline, though not statistically significant, association with ROP development (OR = 11.05; *P* = .053). In contrast, no statistically significant relationships were found between the other factors and ROP development in the multivariate analysis (all*P* > .05) (see Table 3).

No relevant multicollinearity was observed among the variables included in the final multivariable logistic regression model. The variance inflation factor values for LAR2, gestational age, birth weight, mechanical ventilation, RDS, PDA, and surfactant therapy were 1.14, 2.85, 2.37, 1.54, 1.44, 1.24, and 1.59, respectively, all below the conservative threshold of 3. The model showed acceptable fit according to the Hosmer–Lemeshow test (*χ*² = 4.22,*P* = .837), with a Nagelkerke *R*² value of 0.535. Although LAR2 showed a borderline association with ROP development, the wide confidence interval indicated limited precision; therefore, this finding was interpreted cautiously.

## Discussion

This study investigated the connection between the development of ROP and albumin and lactate levels, as well as LAR values obtained from these 2 parameters, in the early and late postnatal periods. In the ROP (+) group, albumin levels were lower, and lactate levels were higher in both time periods. While the difference in lactate levels was not statistically significant, LAR levels favored ROP in both periods, indicating a relationship with the disease (*P* <.05). Studies directly evaluating the association of the lactate/albumin ratio with the occurrence and severity of ROP in premature infants are limited in the existing literature. Thus, by assessing the possible biological significance of the LAR in relation to ROP, this study adds to the body of existing literature.

In recent years, the study of systemic inflammatory, metabolic, and nutrition-related biomarkers in ROP has increased. Previous research has investigated the relationship between ROP development or disease severity and serum lactate levels, the systemic immune-inflammation index. The C-reactive protein/albumin ratio, the platelet-to-lymphocyte ratio, the lymphocyte-to-monocyte ratio, and the neutrophil-to-lymphocyte ratio. These markers have been shown to be related to ROP and to provide indirect information about hypoxic-metabolic stress, systemic inflammation, nutritional reserves, tissue perfusion, and overall neonatal disease burden.^19^^-^^21^ In this context, LAR differs from isolated inflammatory or nutritional indices in that it combines lactate-related hypoxic-metabolic stress with albumin-related inflammatory and nutritional status into a single composite index.

Lactate is produced as a by-product of glucose metabolism, with red blood cells, striated muscle, and brain tissue being its primary sources. Tissue hypoxia may lead to an increase in lactic acid levels in the body. VEGF, on the other hand, is released by retinal pigment epithelium and glial cells under hypoxic conditions. HIF-1 is responsible for the transcriptional regulation of VEGF. Studies have shown that lactate, the end product of glycolysis, increases HIF levels and stabilizes HIF-1, thereby increasing VEGF release from endothelial cells.^22^^,^^23^ It has been reported that hypoxic conditions increase retinal lactic acid levels 7-fold.^24^ Lactate also promotes a proangiogenic environment, stabilising HIF-1α and increasing VEGF receptor 2 (VEGFR2) expression and VEGF-A secretion, as well as endothelial cell migration.^4^ Researchers have observed that lactate enhances the angiogenic potential of VEGF in endothelial cells. Therefore, lactate levels may reflect both cellular metabolic status and actual oxygenation status.^25^

Lactate levels have been shown to be predictive of morbidity and mortality in premature infants.^26^ According to reports, very preterm children with elevated blood lactate levels during the first 7 days of life are more likely to develop ROP, and blood lactate values may also affect its development.^19^ Consequently, high lactate levels during the first few days of life may indicate inadequate perfusion and circulatory dysfunction.^27^ The ROP (+) group in this study had higher lactate levels, but this difference was not statistically significant. Both the ROP (+) and ROP (−) groups showed a decline in lactate levels over time.

Both the inflammatory response and nutritional status are reflected in serum albumin levels. Low albumin levels have been linked to a poor prognosis for preterm infants, including sepsis and death, according to earlier research.^28^ The severe ROP group has been shown to have significantly lower postnatal albumin levels.^20^ In the present study, serum albumin levels assessed during both the early and late periods were significantly lower in the ROP (+) group. However, albumin should be interpreted as a non-specific systemic marker for ROP. Lower albumin levels may reflect impaired nutritional reserves, systemic inflammation, vascular permeability and overall disease burden. In this context, the lower albumin levels observed in the ROP group requiring treatment may indicate a greater systemic disease burden.

Lactate-albumin ratio is a composite index that reflects metabolic and inflammatory status, derived from serum albumin and lactate levels. Evidence indicates that LAR may be useful in tool for predicting clinical outcomes in children. Rather than being confined to a particular disease, In critically ill children, LAR is substantially linked to both mortality and the emergence of MODS when measured in the early stages of the illness.^8^ For patients with necrotising enterocolitis, LAR has been shown to be a valid predictor for in-hospital mortality.^29^ Similarly, among children with severe pneumonia, elevated LAR values have been linked to a higher 28-day intensive care unit mortality rate.^30^

Numerous studies have shown that LAR, rather than lactate or albumin alone, is a better indicator of MODS development and mortality among pediatric patients with severe sepsis or septic shock.^31^^,^^12^ High LAR values at admission among septic pediatric patients treated and admitted to intensive care unit have been linked to impaired microvascular blood perfusion and functional capillary density. In other words, it has been discovered that microcirculatory abnormalities, such as decreased microvascular blood perfusion and impaired capillary capacity, are linked to elevated LAR levels in children with sepsis. The importance of LAR as a potential marker of microcirculatory injury has been emphasized.^32^

The current study evaluated the relationship between ROP development and LAR in premature infants at 2 different time points. This was based on the assumption that LAR could be an indicator of microcirculatory damage. The ROP+ group exhibited substantially higher levels in both the early and late periods. However, LAR values showed limited discriminatory performance in ROC analysis, along with a wide confidence interval and a statistically nonsignificant relationship in the multivariable logistic regression model. Moreover, ROP should be considered to be associated with lower gestational age, reduced birth weight, and a greater burden of neonatal morbidity in developing infants. Therefore, the observed differences in LAR levels may reflect the combined effects of hypoxic-metabolic stress, inflammation, and the overall disease burden associated with prematurity rather than being an independent biological signal specific to ROP.

Correlation analyses revealed a negative relationship between LAR1 and gestational age and birth weight, indicating an association with early postnatal hypoxia, inflammation, and metabolic stress. Conversely, LAR2’s borderline negative relationship with those receiving surfactant therapy can be attributed to the treatment effect and respiratory support processes.

Although there was a trend toward increased albumin levels and decreased lactate levels over time, the consistently high LAR levels in the group that developed ROP, compared to those that did not, suggest ongoing metabolic stress and/or inflammation at a tissue level.

There are some restrictions on this study. Owing to its single-centre, retrospective design, the findings may not be broadly generalisable. Significant variations were found in the ROP (+) and ROP (−) subgroups with regard to birth weight and gestational age several neonatal conditions. Therefore, despite the application of multivariate adjustment, residual confounding factors may have influenced the observed association between LAR and ROP. Serum biomarkers were assessed at only 2 time points, which may not fully reflect the dynamic metabolic and inflammatory changes that occur during the development and progression of ROP. Finally, while excluding aggressive ROP cases was intended to create a more homogeneous study population, this approach might have restricted the findings’ applicability to severe ROP phenotypes, revealing potential selection bias. The multivariate model showed only a marginal and statistically non-significant association, and the ROC analysis demonstrated limited discriminatory performance. Therefore, LAR does not emerge as a standalone screening or diagnostic marker for ROP. Large-scale, multicenter, prospective studies are therefore needed for clinical application.

As a result, LAR may contribute to clinical practice as an auxiliary biomarker, providing a combined assessment of physiological impairment in the albumin-lactate axis, rather than being a strong predictor in its own right. Such markers are important for the development of low-cost, accessible screening strategies in neonatal intensive care.

## Figures and Tables

**Table 1.** Analysis of Demographic Data, Clinical Risk Factors, and Blood Values at 24-Hour and 34-36 Weeks’ Postmenstrual Age in Preterm Infants with and Without Retinopathy of Prematurity

		ROP (+) (n = 76)	ROP (−) (n = 74)	*P*
Gestational age (weeks)	Mean ± SD	27.5 ± 2.1	31.15 ± 2.4	.000*
Birth weight (g)	Mean ± SD	976.8 ± 325.8	1548 ± 495	.000*
Gender (female)	n, %	38 (50.0)	38 (51.4)	.86
RDS	n, %	72 (94.7)	61 (82.4)	.01*
BPD	n, %	56 (73.7)	27 (36.5)	.000*
Mechanical ventilation	n, %	73 (95)	51 (68.9)	.000*
PDA	n, %	36 (47.4)	20 (27)	.01*
NEC		19 (25)	9 (12.2)	.04*
Surfactant therapy	n, %	53 (69.7)	26 (35.1)	.000*
Postnatal systemic steroid therapy	n, %	32 (42.1)	13 (17.6)	.001*
Sepsis	n, %	69 (90.8)	45 (60.8)	.000*
First measurement lactate (mmol/L)	Mean ± SD	3.21 ± 1.53	2.9 ± 1.32	.53
First measurement albumin (g/dL)	Mean ± SD	2.80 ± 0.36	3.17 ± 0.36	.000*
Second measurement lactate (mmol/L)	Mean ± SD	1.58 ± 0.52	1.51 ± 0.64	.14
Second measurement albumin (g/dL)	Mean ± SD	3.04 ± 0.49	3.27 ± 0.42	.003*
First measurement LAR	Mean ± SD	1.16 ± 0.56	0.94 ± 0.44	.03*
Second measurement LAR	Mean ± SD	0.53 ± 0.19	0.47 ± 0.21	.02*

Data are presented as n (%) or mean ± SD.

BPD, bronchopulmonary dysplasia; LAR, lactate/albumin ratio; NEC, necrotizing enterocolitis; PDA, patent ductus arteriosus; RDS, respiratory distress syndrome; ROP, retinopathy of prematurity.

^*^P < .05.

**Table 2.** Comparison of Albumin, Lactate, and Lactate-Albumin Ratio Values According to Retinopathy of Prematurity Subgroups

		No-ROP (n = 74)	ROP-r (n = 44)	ROP-t (n = 32)	*P*
First measurement albumin (g/dL)	Mean ± SD	3.17 ± 0.36	2.89 ± 0.36	2.69 ± 0.33	.000*
Second measurement albumin (g/dL)	Mean ± SD	3.27 ± 0.42	3.16 ± 0.45	2.89 ± 0.49	.000*
First measurement lactate (mmol/L)	Mean ± SD	2.99 ± 1.39	3.33 ± 1.67	3.05 ± 1.32	.76
Second measurement lactate (mmol/L)	Mean ± SD	1.51 ± 0.64	1.57 ± 0.54	1.59 ± 0.48	.29
First measurement LAR	Mean ± SD	0.94 ± 0.44	1.17 ± 0.61	1.14 ± 0.48	.10
Second measurement LAR	Mean ± SD	0.47 ± 0.21	0.51 ± 0.20	0.56 ± 0.17	.02*

Kruskal–Wallis test used.

LAR, lactate/albumin ratio; ROP, retinopathy of prematurity.

^*^P < .05.

**Table 3.** Multivariate Logistic Regression Investigation of Laboratory Data and Clinical Risk Variables Related to the Development of Retinopathy of Prematurity

	B	OR (Exp(B))	*P*
LAR2	2.655	11.05 (%95 CI = 0.97-125.9)	.053
Gestational age	−0.343	0.71 (%95 CI = 0.53-0.94)	.015*
Birth weight	−0.001	1.00 (%95 CI = 0.99-1)	.138
Mechanical ventilation	2.034	7.65 (%95 CI = 0.63-18)	.048*
RDS	−0.627	0.53 (%95 CI = 0.135-6)	.552
PDA	−0.305	0.74 (%95 CI = 0.28-2)	.576
Surfactant therapy	0.326	1.39 (%95 CI = 0.55-4)	.566

BPD, bronchopulmonary dysplasia; LAR, lactate/albumin ratio; NEC, necrotizing enterocolitis; PDA, patent ductus arteriosus; RDS, respiratory distress syndrome.

*P < .05.

## Data Availability

The data that support the findings of this study are available on request from the corresponding author.
